# Quantitative muscle strength assessment in duchenne muscular dystrophy: longitudinal study and correlation with functional measures

**DOI:** 10.1186/1471-2377-12-91

**Published:** 2012-09-13

**Authors:** Alberto Lerario, Serena Bonfiglio, MariaPia Sormani, Andrea Tettamanti, Sarah Marktel, Sara Napolitano, Stefano Previtali, Marina Scarlato, MariaGrazia Natali-Sora, Eugenio Mercuri, Nereo Bresolin, Tiziana Mongini, Giancarlo Comi, Roberto Gatti, Fabio Ciceri, Giulio Cossu, Yvan Torrente

**Affiliations:** 1 Dipartimento di Fisiopatologia medico-chirurgica e dei Trapianti, Centro Dino Ferrari, Department of Neurology and Laboratory of Neuroscience, IRCCS Istituto Auxologico Italiano, Università di Milano, Fondazione, Ospedale Maggiore Policlinico di Milano, Via F. Sforza n35, Milano, 20122, Italy; 2 Biostatistics Unit, Dep. of Health Sciences, University of Genoa, Genoa, Italy; 3 School of Physiotherapy, University Vita-Salute San Raffaele, Milan, Italy; 4 Pediatric Immunohematology and Bone Marrow Transplantation Unit, San Raffaele Scientific Institute, Milan, Italy; 5 Division of Neuroscience, Department of Neurology and INSPE, San Raffaele Scientific Institute, Milan, Italy; 6 Department of Pediatric Neurology, Catholic University, Policlinico Gemelli, Largo Gemelli, 00168, Rome, Italy; 7 Neuromuscular Center "P. Peirolo", Department of Neuroscience, S.Giovanni Battista Hospital of Turin, Turin, Italy; 8 Division of Regenerative Medicine, San Raffaele Scientific Institute, Milan, Italy; 9 Department of Cell and Developmental Biology, University College London, Rm 545, Rockefeller Bldg. 21 University Street, London, WC1E 6DE, UK

## Abstract

**Background:**

The aim of this study was to perform a longitudinal assessment using Quantitative Muscle Testing (QMT) in a cohort of ambulant boys affected by Duchenne muscular dystrophy (DMD) and to correlate the results of QMT with functional measures. This study is to date the most thorough long-term evaluation of QMT in a cohort of DMD patients correlated with other measures, such as the North Star Ambulatory Assessment (NSAA) or thee 6-min walk test (6MWT).

**Methods:**

This is a single centre, prospective, non-randomised, study assessing QMT using the Kin Com^®^ 125 machine in a study cohort of 28 ambulant DMD boys, aged 5 to 12 years. This cohort was assessed longitudinally over a 12 months period of time with 3 monthly assessments for QMT and with assessment of functional abilities, using the NSAA and the 6MWT at baseline and at 12 months only. QMT was also used in a control group of 13 healthy age-matched boys examined at baseline and at 12 months.

**Results:**

There was an increase in QMT over 12 months in boys below the age of 7.5 years while in boys above the age of 7.5 years, QMT showed a significant decrease. All the average one-year changes were significantly different than those experienced by healthy controls. We also found a good correlation between quantitative tests and the other measures that was more obvious in the stronger children.

**Conclusion:**

Our longitudinal data using QMT in a cohort of DMD patients suggest that this could be used as an additional tool to monitor changes, providing additional information on segmental strength.

## Background

Duchenne Muscular Dystrophy (DMD) is an X-linked recessive disorder and is the most common muscular dystrophy in children [1-6] The increasing number of possible therapeutic strategies ready to enter in phase 2 or 3 clinical trials has highlighted the need for reliable and reproducible outcomes measures. There has also been increasing evidence of the need to collect natural history data in order to establish the rate of progression and the variability of muscle strength and functional changes in a disease that does not have a linear progression with increasing age. The North Star Ambulatory Assessment (NSAA) and the 6 min walking test (6MWT), a measure previously used in other disorders, have been recently proposed as possible measures for ambulant DMD [7-9] The choice of this measures reflect the need for assessing aspects of function that are clinically meaningful for patients. In a research setting, however, the trial design may also need objective and sensitive measures of individual muscles, as opposed to functional scales that often measure movements involving many muscles in different muscle groups. The QMT (Quantitative Muscle Testing) is a sensitive tool to measure even small variations of muscle strength, allowing to test specific muscle groups [10-20]. And to monitor the response to treatment of individual muscles or groups of muscles [11,12,16]. Reliability of strength testing with QMT has already been established in healthy controls [13-15,21], and QMT, assessed using the Kin Com^®^ dynamometer, has also already been used in DMD boys in studies on myoblast transplant [11,12].

The aim of our single centre, prospective, non-randomised, study was i) to provide a longitudinal assessment of quantitative muscle strength over one year in a cohort of ambulant DMD boys, ii) to assess possible differences with a small cohort of age- matched male controls and iii) to establish its correlation with other measures such as functional scales and 6MWT .

## Methods

### Study population

28 DMD patients were selected on the basis of a confirmed DMD diagnosis (i.e. clinical features, serum creatine kinase, muscle biopsy and genetic analysis), age between 5 and 12 years, ability to ambulate at the time of selection, absence of severe cardiac and pulmonary disease. Median age was 8.5 years (range 5–12). All had a diagnosis of DMD with clinical findings consistent with DMD, absence of dystrophin on muscle biopsy confirmed by genetic analysis. All but two were on steroids with a median prednisolone adjusted dose of 11.5 mg/kg/month (range 7.5-24). Steroid dosage was not changed during the study but weight adjustment was applied. None of the subjects had health problems other than DMD. Vital signs such as oxygen saturation, respiratory frequency, body temperature and diastolic blood pressure were normal for age. Neurological examination at the time of selection showed anserin ambulation in 25/28 patients and reduced tendon reflexes in 25/28 patients. No one had severe scoliosis or severe contractures that could have limited the use the assessment of strength. Serum chemistry evidenced a median serum creatine kinase of 6408 (range: 3204–26353), AST of 226 U/L (range: 89–629) and ALT of 345 U/L (range 113–702). Baseline cardiological examination, ECG and echo were normal respectively in 28 (100%), 24 (86%), 26 (89%) subjects. 4 subjects showed right bundle branch block and 2 respectively minimal mitral valve insufficiency and posterior wall hypertrophy of no clinical relevance. Pulmonary function tests were evaluable in 25 subjects, normal in 20 subjects while 5 subjects had minimal respiratory insufficiency. This study was approved by the ethical committee of the San Raffaele hospital of Milan (Italy)(reference number DMD01). Parents of all included affected and non-affected boys agreed and signed a written informed consent for the participation in the study.

### Functional and quantitative muscle assessment

The study cohort was assessed at three month intervals for one year for QMT and at baseline at 12 months for a more comprehensive assessment, also using the NSAA and 6MWT. The control group only performed quantitative muscle test assessment at baseline at 12 months.

### Quantitative assessment

Two examiners performed the evaluation using the Kin Com^®^ Robotic Dynamometer (Chattanooga Group Inc., Chattanooga, TN, USA) The muscle groups tested with Kin Com^®^ were knee extensor and flexor, through isometric and isokinetic protocol and elbow extensor and flexor, through isometric protocol. During both lower and upper limb evaluation, subjects seated on the chair of a dynamometer. The seat was adjusted to the size of the boy. A seat belt around the hips and two around the trunk were used to maintain proper position during testing, to isolate the single joint test (knee or elbow) by limiting patient compensatory movements patient during exercise. The boy was asked to keep his arms folded across his chest. The evaluator aligned the axis of the dynamometer to the articular axis of the tested joint (knee or elbow). The lever arm length was adjusted in proportion to the length of the leg (the pad was placed proximal to the ankle joint). During isometric tests knee and elbow joint were positioned at 90°, measured with a goniometer, while during knee isokinetic test the range of motion varied from 90° to 10°. The DMD boy watched the computer screen for visual-feedback during the contraction. The evaluation was divided in two session: the first regarding lower limb lasting 40 min and a second session for the upper limb evaluation which took approximately 15 min. Between sessions patients had at least 30 min of rest while they could come back to their parents. Subjects were asked to perform three times each exercise with a maximum contraction, kept for five seconds. During evaluation the subject was asked to pull or push as hard as he could and the same evaluator gave, for each test, the same continuous verbal encouragements.

### Functional assessment

#### NSAA

This scale, specifically designed for ambulant DMD boys (see Additional file 1: Table S1 in the supplementary information) consists of 17 items, ranging from standing (item 1) to running (item 17) and includes several items assessing abilities that are necessary to remain functionally ambulant, items assessing abilities, such as head raise and standing on heels that can be partly present in the early stages of the disease and a number of activities such as hopping, or running that are generally never fully achieved in untreated DMD boys but that have been found in those treated with daily steroids.

Each item can be scored on a 3 point scale using simple criteria: 2 -Normal achieves goal without any assistance; 1 -Modified method but achieves goal independent of physical assistance from another person; 0 - Unable to achieve independently.

A total score can be achieved by summing the scores for all the individual items. The score can range from 0, if all the activities are failed, to 34, if all the activities are achieved. The scale is generally completed in a maximum of 15 min. The NSAA also includes the possibility to record timed items (10 m timed walk/run test and time to rise from the floor or Gower test) [12,13,22].

#### 6MWT

The 6MWT was performed using a modified version of the American Thoracic Society (ATS) guidelines for the test [7,23]. Modifications include the addition of a short orientation video prior to testing, continuous encouragement from the testing staff, and a “safety chaser” to walk along behind the subject during testing. The test is generally completed within 15 to 20 min, including the instructions. The assessments were performed by examiners specifically trained on this measures. Videotaping of both assessments was recorded after consent was obtained. During 6-min walking test patients were asked to walk steadily and quickly, without running, along a distance of 15 m. Encouragement was given during the assessment, and DMD boys were informed each minute of the time remaining. Patients were allowed to stop, if necessary, and start again within the 6 min.

### Statistical analysis

Kin Com^®^ measurements were evaluated cross-sectionally and longitudinally over a 12 months period of time with 3 monthly assessments. Left and right measurements were averaged and the mean value was used for all the analyses. Differences between baseline values and those measured after one year between patients and control subjects were compared by an ANOVA model, adjusting for age. In the patients’ group, the dependence of the baseline values with age was preliminarily assessed by a visual inspection of the plots and non-linear relationships with age were analysed using a piecewise linear regression. A piecewise regression model allows for changes in slope and consists of two or more straight line segments. Models with one point of slope change were fitted. The time trend of the Kin Com^®^ measures over one year was assessed using a mixed effect linear model accounting for the within-subjects correlations, with a time x age interaction term, evaluating whether the slope of change is different across ages. Different age cut off points were used to categorize age in the interaction analysis (as above and below the cut off point). Correlations between baseline values and longitudinal slopes were evaluated using a Spearman rank correlation coefficient. The quantification of the random fluctuations of the Kin Com^®^ variables of an untreated population is important to understand whether a change over time in a clinical trial can be considered within fluctuations that can be expected by chance or it’s large enough to be considered due to a treatment effect. Therefore the Kin Com^®^ variations at three months intervals were studied: a regression line was fitted for each patient and a linear trend of the Kin Com^®^ variables over time was estimated. An analysis of the residuals (distances of the observed values from the predicted regression lines) was used to estimate the magnitude of the random fluctuations of each Kin Com^®^ variable. Random fluctuations were defined as those within the 95% Confidence Intervals of the distributions of residuals. All the statistical analyses were conducted using SPSS vers 18.0 and R vers.2.11.1.

## Results

### Descriptive analysis

The baseline characteristics of the studied population are reported in Table 1. The average age is 8.3 years (SD = 2.3), with 8 subjects in the range 5–7, 13 subjects in the range 7–9 and 7 subjects in the range 9–12.

**Table 1:** Baseline characteristics of the enrolled population

	**Patients N = 28**	**Controls N = 13**	**P value**
	**Mean (SD) Median (range)**		
Age	8.4 (1.6) 8.5 (5.6-12.0)	9.5 (2.8) 10.5 (5.5-12.5)	0.11
NS	24.2 (8.5) 27.5 (4–34)	-	-
6MWT (m)	361.2 (98.0) 386 (42–518)	-	-
10 M (s)	6.8 (6.3) 5.1 (3–36)	-	-
GOWERS (s)	8.0 (9.5) 4.7 (1–45)	-	-
Isometric KE (N)	56 (35) 53 (10–134)	310 (151) 251 (135–628.3)	<0.01*
Isometric KF (N)	52 (20) 51 (14–93)	130 (55) 124 (60–225)	<0.01*
Isocinetic KE (N)	59 (33) 51 (15–141)	276 (128) 260 (130–546)	<0.01*
Isocinetic KF (N)	48 (20) 49 (10–100)	124 (46) 122 (59–223)	<0.01*
EE (N)	26 (14) 24 (5–56)	101 (49) 94 (25–168)	<0.01*
EF (N)	24 (10) 22 (7–46)	113 (57) 100 (55–225)	<0.01*

*p values adjusted for age.

Abbreviations: NS = North Star Scale; 6MWT = 6-min walking test; KE = Knee Extension; KF = Knee Flexion; EE = Elbow Extension; EF = Elbow Flexion.

### Baseline values

**Figure 1:**
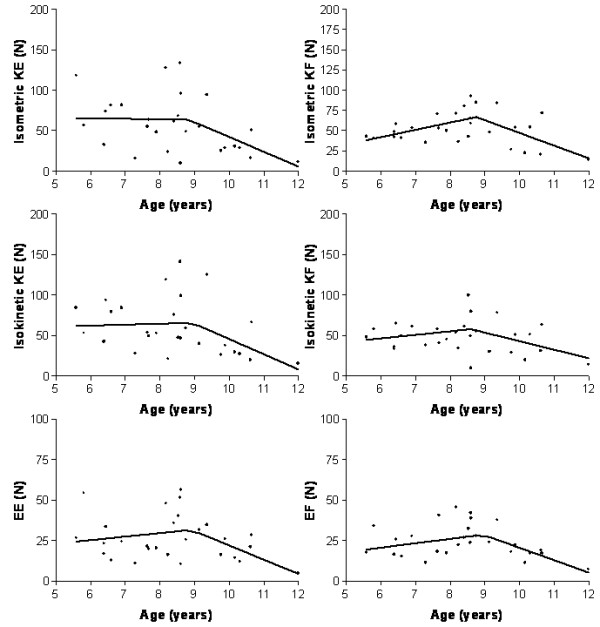
**Baseline values of the Kin Com^®^ measures are plotted against patients age.** The fitted lines represent the linear dependency of these values with age, with one point of slope change, that was estimated by a piece-wise regression.

Average baseline values of the functional scales and Kin Com^®^ measures for patients and healthy controls are reported in Table 1. All the Kin Com^®^ measurements were significantly lower in patients than in controls. The relationships of the patients’ measurements with age were analysed by a linear model and by a piecewise linear regression model with one point of slope change. The piecewise regression model resulted better fitting the relationships between all the baseline values of the functional scales than a simple linear regression model (Figure 1). The results of the piecewise regression analysis are reported in Additional file 1: Table S1 for all the Kin Com^®^ measures the point of slope change was around 8.5 years. The coefficients reported in Additional file 1: Table S1 indicate an increase with age when positive and a decrease with age when negative: for example, before the age of 8.8 years isometric KF was the measurement with the highest increase with age (+9 points per year, p = 0.017) while after the age of 8.8 years it showed significant decrease of −15.7 points per year (p = 0.0015, Figure 1).

### Baseline correlations

**Figure 2:**
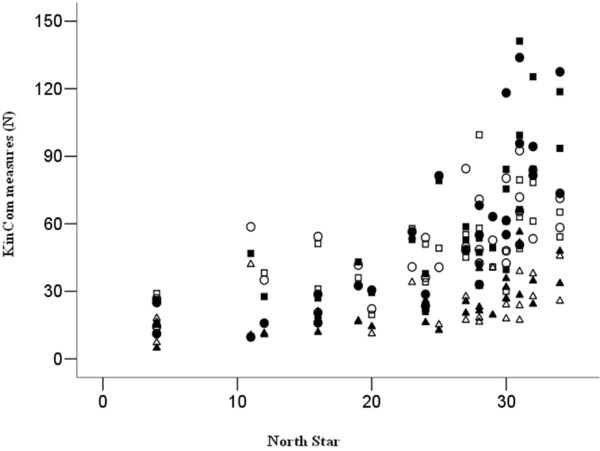
**Correlations between all the Kin Com^®^ measures with the North Star functional scale at baseline.** Each symbol represent a different Kin Com^®^ variable: Black dot: Isometric Knee Extension, White dot: Isometric Knee Flexion, Black square: Isocinetic Knee Extension, White square: Isocinetic Knee Flexion, Black triangle: Elbow Extension, White triangle: Elbow Flexion.

Table 2 reports the correlations between baseline values of 6MWT, NSAA and the Kin Com^®^ measures. All the baseline Kin Com^®^ measures values highly correlate with all the other measures. Correlation of the baseline Kin Com^®^ values with the baseline NS scores is shown in Figure 2.

**Table 2:** Baseline correlations

		**NS**	**6MWT (m)**	**TIME 10 m (s)**	**Gowers (s)**	**Isometric KE (N)**	**Isometric KF (N)**	**Isocinetic KE (N)**	**Isocinetic KF (N)**	**EF (N)**
6MWT (m)	r	0.80	1							
	p	<0.001								
TIME10m (s)	r	−0.93	−0.80	1						
	p	<0.001	<0.001							
Gowers (s)	r	−0.89	−0.75	0.91	1					
	p	<0.001	<0.001	<0.001						
Isometric KE (N)	r	0.86	0.70	−0.89	−0.87	1				
	p	<0.001	<0.001	0.01	<0.001					
Isometric KF (N))	r	0.66	0.60	−0.64	−0.52	0.50	1			
	p	<0.001	<0.001	<0.001	0.007	<0.001				
Isocinetic KE (N)	r	0.87	0.72	−0.86	−0.88	0.93	0.64	1		
	p	<0.001	<0.001	0.01	<0.001	<0.001	<0.001			
Isocinetic KF (N)	r	0.64	0.63	−0.68	−0.66	0.62	0.73	0.70	1	
	p	<0.001	<0.001	<0.001	<0.001	<0.001	<0.001	<0.001		
EF (N)	r	0.63	0.68	−0.65	−0.63	0.63	0.52	0.55	0.59	1
	p	<0.001	<0.001	.01	0.001	<0.001	<0.001	<0.001	<0.001	
EF (N)	r	0.75	0.59	−0.84	−0.77	0.75	0.55	0.73	0.65	0.76
	p	<0.001	<0.001	0.01	<0.001	<0.001	<0.001	<0.001	<0.001	<0.001

Abbreviations: r = Pearson correlation coefficient, NS = North Star Scale; 6MWT = 6-min walking test; KE = Knee Extension; KF = Knee Flexion; EE = Elbow Extension; EF = Elbow Flexion.

### Longitudinal assessment

Additional file 2: Table S2 reports the one-year changes of all the Kin Com^®^ measurements in patients and in healthy controls. All the average one-year changes of patients were significantly lower than those experienced by healthy controls (Additional file 2: Table S2), who showed as expected a progressive improve. Additional file 3: Table S3 reports the results of the longitudinal analysis in the patients group. As for baseline values, most of the longitudinal changes (excluding EE and EF) of the Kin Com^®^ measurements depend on age; this is represented by a significant age x time interaction in Additional file 3: Table S3. For Kin Com^®^ measures with a time trend significantly different at different ages (age x time interaction), the slope (representing the average change for a three months interval) is reported separately for children above and below the age of 7.5 years. This value resulted as the cut off better discriminating one-year changes in this population. This is lower than the value of 8.5 years that was estimated by the cross-sectional analysis; however, the small sample of patients used in this analysis justifies a certain degree of variability in detecting the age of slope change when analyzed with different approaches. On the average, all the Kin Com^®^ measures increase their value over the 12 months of observation in children below the age of 7.5 years: isometric KE has a 3 monthly significant increase of 2.4 points (SE = 0.8, p < 0.001) and isometric KF of 1.0 point (SE = 0.3, p = 0.002). Isokinetic KE and KF also increased in children below 7.5 years, but this change was not statistically significant. EE and EF show a significant increase over time for the whole cohort of children (+1.2 (SE = 0.1) and +0.6 (SE = 0.1) points every 3 months, p < 0.001 for both).

In children above the age of 7.5 years, isometric KE, isokinetic KE and isokinetic KF showed a significant decrease (−0.4 (SE = 0.2), -0.4 (SE = 0.2), and −0.6 (SE = 0.2) points every 3 months, p = 0.04, p = 0.04 and p < 0.001 respectively). All the average one-year changes were significantly different than those experienced by healthy controls (Additional file 2: Table S2).

### Correlations of longitudinal changes

**Figure 3:**
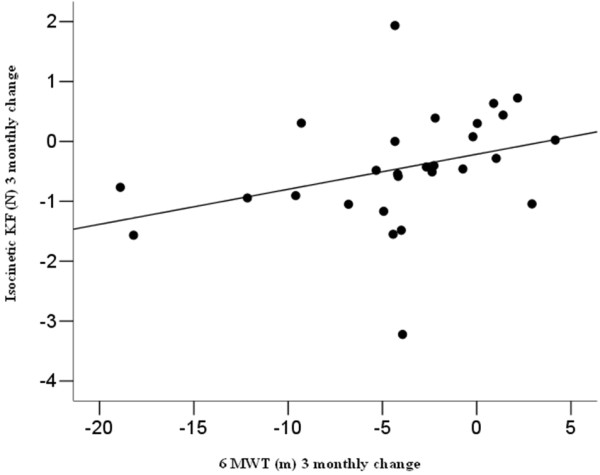
The 3 monthly change of and isocinetic KF is plotted against the 3 monthly change of the 6MWT.

In Additional file 3: Table S3 the correlations between the one-year change, expressed as a 3 monthly slope, of Kin Com^®^ measures and functional scales are reported*.* The 3 monthly change (Figure 3) of the isometric KE values correlated with the change of the NS values (r = 0.32, p = 0.09) and of the GOWERS values (r = −0.40, p = 0.04); the 3 monthly change of the isometric KF correlated with the change of the 6MWT (r = 0.50, p = 0.01) and the change of the 10 m time (r = −0.47, p = 0.01); the 3 monthly change of the isokinetic KF correlated with the change of the 6MTW (r = 0.51, p = 0.01) (Figure 3).

### Estimation of random fluctuations over time

In order to estimate the random fluctuations of the Kin Com^®^ values, the residuals around the linear trend over time for each patient were examined (Additional file 4: Table S4). A regression line was estimated for each patient according to the 3-monthly spaced time points (months 0-3-6-9-12) and the deviations from these regression lines were estimated and mediated over all the patients. As reported in Additional file 4: Table S4, if a slope is estimated for a patient, deviations between the 9% and the 17% of the baseline value can be observed. In absolute terms, deviations of 12.5 N for Isometric KE, of 9.6 N for Isometric KF, of 10.2 N for Isocinetic KE and Isocinetic KF, of 7.8 N for EE and of 6.9 N for EK can be considered random fluctuations around each patient’s slopes.

## Discussion

In the last few years there have been large international efforts in order to identify outcome measures in DMD. The course of DMD is actually measured by serial clinical assessments of muscle strength, pulmonary function, and functional rating scales. A number of measures including the 6MWT and different timed items or functional scales have been proposed for ambulant DMD patients as part of safety studies or in early stages of treatment. These measures rely on patient effort or on subjective rating of function. However some treatments may be administered to specific groups of muscles, and we therefore need additional measures that may detect and monitor minimal changes in a single muscle or in muscle groups. For these reasons, quantitative assesment have been explored as adjuncts to the physical examination in the assessment of patients with neuromuscular disorders. In this study we assessed QMT using the Kin Com^®^, an assessment already used in DMD boys in previous studies and with pediatric normative data. The results of our study show that this technique can be reliably used in DMD boys and that can assess changes over time. All our DMD boys were able to understand instructions to perform the quantitative assessments, showing good participation and motivation. Furthermore, although two patients lost ambulation in the course of the year and thus were unable to continue to perform functional measures, all patients completed the assessment with the Kin Com^®^ dynamometer. Having five time points (3 monthly spaced assessments over one year) we were also able to estimate a linear trend with time for each Kin Com^®^ variable. The deviations from these trends represent the normal random fluctuations that can be expected in a population of DMD patients [22,24-29]. It is of interest that the results of both cross sectional and longitudinal assessment showed that the slope of deterioration in DMD boys occurs at approximately 7.5 years. These results are in agreement with our recent observation obtained in a multicentric study and in a much larger cohort using 6MWT and NSAA. Not surprisingly, the decline was more obvious after the age of 9, in all quantitative tests performed with the Kin Com^®^ dynamometer during right and left isometric and isokinetic flexion/extension of the knee and during isometric flexion/extension of the elbow. The strength value of lower and upper limbs obtained with Kin Com^®^ show no significant difference between right and left lower limbs or between right and left upper limbs. All the average one-year changes were significantly different than those experienced by the age matched controls. In order to have a more complete assessment, at baseline and 12 months the study cohort was also assessed using the NSSA and the 6MWT. There was a high degree of correlation between the functional tests and isokinetic knee extension tests, especially for correlation between isokinetic knee tests and value of North Star scale.

The results obtained indicate that the use of QMT, in addition to the functional scales, provides sensitive and objective information of muscle strength changes of DMD patients, suggesting that the Kin Com^®^ dynamometer is a valid, sensible and reproducible tool to evaluate muscle strength in ambulant and allowed us to have some results also in patients who lost ambulation after baseline. Therefore, the combination of isometric/isocinetic QMT and functional measures should be regarded as a useful outcome measure for clinical trials in which the mechanism of the drug is expected to produce an increase in strength. Although the numbers were relatively small, our findings information expand the spectrum of our knowledge on individual measures but also provides more insights on their correlation as quantitative muscle strength assessments, 6MWT and functional measures have all been previously investigated in DMD but had never been assessed in the same cohort.

## Conclusion

Prospective, longitudinal multicenter studies are needed to assess the relative sensitivity of isometric/isocinetic QMT to measure changes in DMD disease pathology as compared to functional strength outcome measures and for detecting the effects of future treatments. Depending on the design of the trial, the combination of functional measures and Kin Com^®^ dynamometer assessment may offer a more complete and sensitive method of outcome measures when objective measures of strength are needed.

### Previous presentation

Presented as oral communication at 39th European Muscle Conference of the European Society for Muscle Research. Abano Terme, Padova, Italy, September 11–15th, 2011.

## Competing interests

The authors declare that they have no competing or financial interests.

## Authors’ contributions

GC, FC, YT conceived of the study, participated in its design and coordination, and corrected the manuscript draft. AL, SB, MPS, YT carried out the analysis, data collection and wrote the manuscript. A S C P, M S, M G N S. performer neurological examination. AT, SM, SN, SCP, MS, MG NS, NB, G C, RG. helped with the sampling and data collection process. All authors read and approved the final manuscript. Statistical analysis was performed by MP S.

## Funding

Duchenne Parent Project, International Collaborative Effort for DMD (ICE), EC 7 Eventh Framework Progrmme IP 223098 (Optistem), Province of Trento, Fondazione Telethon.

## Pre-publication history

The pre-publication history for this paper can be accessed here:

http://www.biomedcentral.com/1471-2377/12/91/prepub

## Supplementary Material

Additional file 1 **Table S1.**Relationship of patients’ baseline values of the KinCom variables with age. Click here for file

Additional file 2 **Table S2.**1 year follow up of DMD patients and healthy subjects. Click here for file

Additional file 3 **Table S3.**1 year longitudinal changes of patients’ KinCom variables according to age. Click here for file

Additional file 4 **Table S4.**Fluctuations of the KinCom variables around the regression lines over 3 months of intervals. Click here for file
