# Supplementary material for: Quantitative muscle strength assessment in duchenne muscular dystrophy: longitudinal study and correlation with functional measures
Source: BMC Neurol. 2012 Sep 13;12:91. doi: 10.1186/1471-2377-12-91 (PMC3482602; doi:10.1186/1471-2377-12-91)
Supplement: Additional file 1 — Table S1.Relationship of patients’ baseline values of the KinCom variables with age. [file 1471-2377-12-91-S1.doc]

**Supplemental Table 1**: relationship of patients’ baseline values of the KinCom variables with age.

|  | Variables | Coefficients | p values |
| --- | --- | --- | --- |
| **Isometric KE (N)** | Age [<8.8]*  Age [≥8.8]* | −0.5 (6.7)  −19.0 (10.7 ) | 0.94  0.18 |
| **Isometric KF (N)** | Age [<8.7]*  Age [≥8.7]* | +9.1 (3.5)  −15.7 (5.5) | 0.017  0.0015 |
| **Isocinetic KE (N)** | Age [<8.9]*  Age [≥8.9]* | +1.2 (6.7)  −18.7 (11.2) | 0.85  0.18 |
| **Isocinetic KF (N)** | Age [<8.6]*  Age [≥8.6]* | +4.4 (3.8)  −10.5 (5.5) | 0.25  0.04 |
| **EE (N)** | Age [<8.9]*  Age [≥8.9* | +2.2 (2.7)  −8.6 (4.4) | 0.41  0.06 |
| **EF (N)** | Age [<8.9]*  Age [≥8.9]* | +2.7 (0.9)  −7.6 (2.9) | 0.13  0.009 |

Abbreviations: NS=North Star Scale; 6MWT=6-minute walking test; KE=Knee Extension; KF=Knee Flexion; EE=Elbow Extension; EF=Elbow Flexion
