# Supplementary material for: Quantitative muscle strength assessment in duchenne muscular dystrophy: longitudinal study and correlation with functional measures
Source: BMC Neurol. 2012 Sep 13;12:91. doi: 10.1186/1471-2377-12-91 (PMC3482602; doi:10.1186/1471-2377-12-91)
Supplement: Additional file 2 — Table S2.1 year follow up of DMD patients and healthy subjects. [file 1471-2377-12-91-S2.doc]

**Supplemental Table 2:** 1 year changes in DMD patients and healthy controls

|  | **Patients**  **N=28** | **Controls**  **N=13** | **P value** |
| --- | --- | --- | --- |
|  | **Mean (SD)**  **Median (range)** | |  |
| **Isometric KE change (N)** | 3 (30)  −2 (−34,104) | 74 (70)  61 (−16,194) | <0.01* |
| **Isometric KF change (N)** | 5 (15)  5 (−25,32) | 28 (25)  24 (2,79) | <0.01* |
| **Isocinetic KE change (N)** | −4 (20)  −5 (−47,45) | 18 (41)  4 (−45,80) | 0.03* |
| **Isocinetic KF change (N)** | −4 (13)  −5 (−48,25) | 11 (28)  7 (−36,55) | <0.01* |
| **EE change (N)** | 13 (12)  11 (−6,47) | 32 (38)  24 (−12,104) | 0.05* |
| **EF change (N)** | 6 (10)  5 (−21,33) | 33 (19)  37 (7,62) | <0.01* |

*p values adjusted for age

Abbreviations: NS=North Star Scale; 6MWT=6-minute walking test; KE=Knee Extension; KF=Knee Flexion; EE=Elbow Extension; EF=Elbow Flexion
