# Supplementary material for: Quantitative muscle strength assessment in duchenne muscular dystrophy: longitudinal study and correlation with functional measures
Source: BMC Neurol. 2012 Sep 13;12:91. doi: 10.1186/1471-2377-12-91 (PMC3482602; doi:10.1186/1471-2377-12-91)
Supplement: Additional file 3 — Table S3.1 year longitudinal changes of patients’ KinCom variables according to age. [file 1471-2377-12-91-S3.doc]

**Supplemental Table 3**: longitudinal one year changes of patients’ KinCom variables according to age

| **Variable** | **Isometric KE (N)** | | **Isometric KF (N)** | | **Isocinetic KE (N)** | | **Isocinetic KF (N)** | | **EE (N)** | **EF (N)** |
| --- | --- | --- | --- | --- | --- | --- | --- | --- | --- | --- |
| **Time x age interaction** | <0.001 | | 0.04 | | 0.01 | | 0.03 | | 0.8 | 0.6 |
| **Age** | <7.5 | >=7.5 | <7.5 | >=7.5 | <7.5 | >=7.5 | <7.5 | >=7.5 | all | all |
| **slope** | +2.4 (0.8) | −0.4 (0.2) | +1.0 (0.3) | +0.3 (0.2) | +0.6 (0.4) | −0.4  (0.2) | +0.1 (0.3) | −0.6  (0.2) | +1.2 (0.1) | +0.6 (0.1) |
| **P value** | 0.005 | 0.04 | 0.002 | 0.16 | 0.14 | 0.04 | 0.7 | <0.001 | <0.001 | <0.001 |

Abbreviations: KE=Knee Extension; KF=Knee Flexion; EE=Elbow Extension; EF=Elbow Flexion
