# Supplementary material for: Quantitative muscle strength assessment in duchenne muscular dystrophy: longitudinal study and correlation with functional measures
Source: BMC Neurol. 2012 Sep 13;12:91. doi: 10.1186/1471-2377-12-91 (PMC3482602; doi:10.1186/1471-2377-12-91)
Supplement: Additional file 4 — Table S4.Fluctuations of the KinCom variables around the regression lines over 3 months of intervals. [file 1471-2377-12-91-S4.doc]

**Supplemental Table 4: Fluctuations of the KinCom variables around the regression lines describing the time course and estimated over 3 months intervals**.

|  | Min  Deviation (N) | Max deviation (N) | Average SD (N) | COV (referred to baseline value) | Random fluctuations range (N) |
| --- | --- | --- | --- | --- | --- |
| **Isometric KE** | 0.53 | 21.10 | 6.4 | 11% | ±12.5 |
| **Isometric KF** | 0.77 | 10.81 | 4.9 | 9% | ±9.6 |
| **Isocinetic KE** | 0.29 | 14.07 | 5.2 | 9% | ±10.2 |
| **Isocinetic KF** | 1.07 | 16.16 | 5.2 | 11% | ±10.2 |
| **EE** | 0.56 | 10.76 | 4.0 | 17% | ±7.8 |
| **EK** | 0.75 | 7.83 | 3.5 | 13% | ±6.9 |

Min, Max deviations = Minimum and maximum deviation from the linear trend estimated for each patient. Average SD = Average value of the standard deviations (SD) of the distribution of residuals for each patient. For each patient the SD of the distribution of residuals represents the average value of all the random fluctuation around the linear trend estimated for each patient using the 3 months intervals value of the Kin Com® variables.

COV (referred to baseline value) = ratio between the average SD and the average baseline value of each Kin Com® variable. Random fluctuations range = values obtained as 1.96*Average SD. These values represent the normal range (at a confidence level of 95%) of fluctuation around the slope over time of the Kin Com® variables in an untreated population of subjects.
